# Investigation of Atmospheric Effects on Retrieval of Sun-Induced Fluorescence Using Hyperspectral Imagery

**DOI:** 10.3390/s16040480

**Published:** 2016-04-06

**Authors:** Zhuoya Ni, Zhigang Liu, Zhao-Liang Li, Françoise Nerry, Hongyuan Huo, Rui Sun, Peiqi Yang, Weiwei Zhang

**Affiliations:** 1 State Key Laboratory of Remote Sensing Science, School of Geography, Beijing Key Laboratory of Environmental Remote Sensing and Digital City, Beijing Normal University, Beijing 100875, China; nizhuoya1987@163.com (Z.N.); sunrui@bnu.edu.cn (R.S.); zhangww0510@163.com (W.Z.); 2 ICube, CNRS, Université de Strasbourg, 300 Boulevard Sébastien Brant, CS10413, Illkirch 67412, France; lizl@unistra.fr (Z.-L.L.); f.nerry@unistra.fr (F.N.); 3 Key Laboratory of Agri-informatics, Ministry of Agriculture/Institute of Agricultural Resources and Regional Planning, Chinese Academy of Agricultural Sciences, Beijing 100081, China; 4 College of Urban and Environmental Sciences, Tianjin Normal University, Tianjin 300387, China; huohongyuan2008@163.com; 5 ITC-Faculty of Geo-Information Science and Earth Observation, University of Twente, Enschede 7514AE, The Netherlands; p.yang@utwente.nl

**Keywords:** sun-induced fluorescence, sensitivity analysis, oxygen-absorption depth, FLD-like method, DOAS, airborne data

## Abstract

Significant research progress has recently been made in estimating fluorescence in the oxygen absorption bands, however, quantitative retrieval of fluorescence data is still affected by factors such as atmospheric effects. In this paper, top-of-atmosphere (TOA) radiance is generated by the MODTRAN 4 and SCOPE models. Based on simulated data, sensitivity analysis is conducted to assess the sensitivities of four indicators—depth_absorption_band, depth_nofs-depth_withfs, radiance and Fs/radiance—to atmospheric parameters (sun zenith angle (SZA), sensor height, elevation, visibility (VIS) and water content) in the oxygen absorption bands. The results indicate that the SZA and sensor height are the most sensitive parameters and that variations in these two parameters result in large variations calculated as the variation value/the base value in the oxygen absorption depth in the O_2_-A and O_2_-B bands (111.4% and 77.1% in the O_2_-A band; and 27.5% and 32.6% in the O_2_-B band, respectively). A comparison of fluorescence retrieval using three methods (Damm method, Braun method and DOAS) and SCOPE Fs indicates that the Damm method yields good results and that atmospheric correction can improve the accuracy of fluorescence retrieval. Damm method is the improved 3FLD method but considering atmospheric effects. Finally, hyperspectral airborne images combined with other parameters (SZA, VIS and water content) are exploited to estimate fluorescence using the Damm method and 3FLD method. The retrieval fluorescence is compared with the field measured fluorescence, yielding good results (*R*^2^ = 0.91 for Damm *vs.* SCOPE SIF; *R*^2^ = 0.65 for 3FLD *vs.* SCOPE SIF). Five types of vegetation, including ailanthus, elm, mountain peach, willow and Chinese ash, exhibit consistent associations between the retrieved fluorescence and field measured fluorescence.

## 1. Introduction

Solar-induced chlorophyll fluorescence (SIF) is emitted by chlorophyll α of vegetation under excitation by solar radiation. Remote sensing of SIF is thought to be a direct, rapid, effective and noninvasive technology to measure global photosynthetic activity. In 1988, Buschmann found that fluorescence signals influence the shapes of reflection spectra [1]. Since then, many studies of the remote sensing of fluorescence have been conducted on the leaf [2,3], canopy [4,5,6,7], airborne [8,9,10,11] and space-borne scales [12]. Much work on SIF retrieval has been performed in the laboratory and in field experiments [8,9,13,14,15,16,17,18,19,20,21,22,23].

Because fluorescence signal intensities are small compared to reflected solar radiation (approximately 2%–5% in the near-infrared region), decoupling fluorescence radiation from reflected radiation is challenging. Measurement of SIF from the ground and air is feasible because SIF constitutes a higher proportion of the signal within the Fraunhofer lines and atmospheric absorption bands and is less affected by atmospheric effects [24]. However, determining fluorescence from space is influenced by atmospheric effects. Thus, researchers’ attention has been focusing on how to remove atmospheric effects and improve the accuracy of SIF retrieval.

In recent years, many methods have been developed to measure SIF from space. Based on the existing literature, methods of retrieving SIF from space always exploit atmospheric absorption features, primarily the oxygen absorption band or Fraunhofer lines. The oxygen absorption band is relatively broad, deep and close to the two characteristic peaks of the fluorescence emission spectrum at 680 and 740 nm. It is thought to be the best candidate band to retrieve SIF [25], and the accuracy of the atmospheric correction can enable high-accuracy SIF retrieval. Methods such as FLD [26], 3FLD [27], cFLD [27,28] and SFM [23] have been developed for use with space measurements combined with the atmospheric parameters computed by MODTRAN [4,12,29,30]. Guanter *et al.* estimated SIF based on oxygen absorption features using atmospheric corrections based on the MODTRAN code to avoid uncertainties caused by atmospheric absorption and scattering effects. They tested this method on Medium Resolution Imaging Spectrometer (MERIS) images and FLEX-like high-resolution data [12,29]. Frankenberg *et al.* have shown an efficient alternative fluorescence least-square retrieval method based on the O_2_-A band, with decoupling of fluorescence from scattering properties, and have applied this method to GOSAT and OCO-2 images [31]. Damm *et al.* have used the FLD method combined with MODTRAN-4 simulated at-sensor radiances to derive canopy chlorophyll fluorescence [11]. They exploited a semi-empirical approach to estimate SIF using the 3FLD method, focusing on atmospheric oxygen absorption bands and using non-fluorescent surfaces to remove atmospheric effects [30]. Joiner *et al.* have used principal components to estimate the spectral structure of atmospheric absorption, and the atmospheric information was incorporated into a simplified radiative transfer model to estimate SIF [32]. Braun has compared the radiation ratio of the O_2_-A band for vegetated and non-vegetated regions in the same image to estimate SIF from Hyperion images [33]. Further, Liu *et al.* have proposed a new PCA-based full-spectrum spectral fitting method (F-SFM) for the retrieval of SIF [34].

Fraunhofer lines can be used to disentangle fluorescence emissions from scattering effects. Joiner *et al.* have assessed the filling in of the potassium K I solar Fraunhofer line near 770 nm and the Ca II line at 866 nm to derive chlorophyll fluorescence from GOSAT images [24,35] and SCIAMACHY [36]. Guanter *et al.* have used a linear forward model derived by a singular vector-decomposition technique in a narrow window containing only Fraunhofer lines to determine inverse SIF from GOSAT images [36,37]. Köhler *et al.* have applied a linear method for the retrieval of SIF from GOME-2 and SCIAMACHY data [38] and have then proposed a simplified physically based fluorescence-retrieval method in a spectral range of 755–759 nm based on GOSAT data [39].

Measurement of SIF from space can provide an important direct approach for diagnosing vegetation stress associated with reduced photosynthetic functionality and for estimating global gross primary productivity. The Fluorescence Explorer (FLEX) was selected for an Earth-exploration mission in November 2015. It will map vegetation fluorescence to quantify photosynthetic activity and will thus provide better insights into plant health and stress [40].

The purpose of this paper is to assess atmospheric effects on SIF retrieval in the O_2_-A band. Compared with Fraunhofer lines, oxygen absorption bands are used more extensively because of their width and depth, but the atmospheric effects on oxygen absorption bands can result in an erroneous estimation of SIF [30]. In this paper, we use SCOPE and MODTRAN 4 to generate simulated top-of-atmosphere (TOA) data, and we evaluate the sensitivity of atmospheric parameters, including the SZA, sensor height, elevation, VIS and water content, to the accuracy of SIF retrieval. In the last part, SIF is retrieved from a hyperspectral image acquired by an AISA sensor with atmospheric parameters to confirm the conclusions of simulation analysis.

## 2. Materials and Methods

### 2.1. Generation of Simulated Data

In this paper, the MODTRAN 4 and SCOPE models are used to simulate TOA radiation, including fluorescence radiation. Assuming that SIF and reflectance follow Lambert’s law, the radiance at TOA, including SIF radiance, can be described as follows:

(1) $$L_{\text{TOA}}=\frac{E_{0}\cos\theta }{\pi }\rho_{\text{so}}+\frac{E_{0}\cos\theta }{\pi }\frac{\left(\tau_{\text{ss}}{+\tau }_{\text{sd}}\right)R\left(\tau_{\text{do}}{+\tau }_{\text{oo}}\right)}{1-R\;\rho }+\frac{\text{SIF}\left(\tau_{\text{do}}{+\tau }_{\text{oo}}\right)}{1-R\;\rho }$$

On the right side of this equation, the first item is the atmospheric contribution to the TOA radiance signal, the second item is the surface-reflected radiance, and the last item is the fluorescence signal contribution to the TOA radiance. In the equation, ρ_so_ is the hemispherical reflectance, $E_{0}$ is the extraterrestrial solar irradiance on a plane perpendicular to the sun’s rays, θ is the solar zenith angle, R is the surface reflectance, SIF is the fluorescence radiance at the top-of-the-canopy (TOC), and ρ is the spherical reflectance of the atmosphere back to the surface, τ stands for transmittance. The first subscript indicates incident radiation, and the second subscript indicates scattered radiation. τ_ss_ + τ_sd_ is the total irradiance transmittance (including direct and diffuse components), and τ_do_ + τ_oo_ is the spherical transmittance from the surface to the TOA. To τ_ss_, the incident and scattered radiation are direct solar radiation; to τ_sd_, the incident radiation is direct solar radiation, and scattered radiation is hemispherical diffuse radiation; to τ_do_, the incident radiation is diffuse sky radiation, and the scattered radiation is the direct radiation in the observer’s direction; to τ_oo_, the incident radiation is direct radiation, and the scattered radiation is the direct radiation in the observer’s direction.

In Equation (1), SIF and R come from the simulation of the SCOPE model, and the other parameters are computed by MODTRAN. The atmospheric radiative transfer code MODTRAN [41] is applied to extract the atmospheric spectral transfer functions for the forward modeling of TOA [42]. To generate the database, 1080 MODTRAN cases (Table 1), including four sun zenith angles, four sensor heights, three elevations, five surface meteorological ranges and three vertical water contents [11,30,32,43,44], are simulated. Every case covers 10,000–25,000 cm^−1^ at a 1-cm^−1^ resolution. The atmospheric parameters, including $\rho_{\text{so}}$, ρ, $\tau_{\text{ss}}$, $\tau_{\text{sd}}$ and $\tau_{\text{do}}$, are computed from the simulated data.

The SCOPE model can simulate fluorescence and reflectance on the canopy using the radiation transfer module and the leaf biochemical model. The input parameters of SCOPE are introduced in detail in the SCOPE documentation and in a paper by Ni *et al.* [45]. In this study, based on the works of Damm *et al.* [30], Liu *et al.* [44] and Daumard *et al.* [43], the chlorophyll *α+b* content (Cab), fluorescence quantum yield efficiency (Fqe) and leaf area index (LAI) are set varied (Table 2). The other parameters are set to the default values. For each atmospheric condition, 48 SCOPE cases are simulated for the generation of reflectance and fluorescence on the canopy. In the final simulation, 43,200 cases are generated.

**Figure 1:**
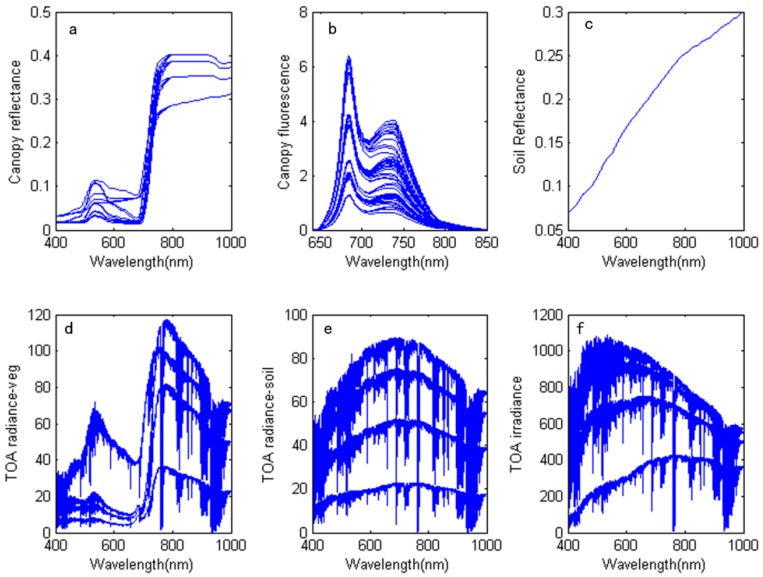
Input spectrum and the simulated results: (**a**) Canopy reflectance spectrum from SCOPE; (**b**) Canopy fluorescence spectrum from SCOPE (W/m^2^/µm/sr); (**c**) Soil reflectance spectrum from the library of ENVI; (**d**) Simulated vegetation TOA radiance (W/m^2^/µm/sr); (**e**) Simulated soil TOA radiance (W/m^2^/µm/sr); and (**f**) Solar irradiance (W/m^2^).

Figure 1 shows the results of the simulated data generation process. Figure 1a–c show the canopy reflectance, fluorescence and soil spectra, respectively. The reflectance and fluorescence spectra are derived from the SCOPE model, and the soil spectrum is derived from the spectral library of ENVI. The reflectance, fluorescence and soil spectra are inputs into the radiative transfer equation to compute the TOA radiance. Figure 1e–g show the simulated results regarding the vegetation radiance, including the fluorescence radiance, soil radiance and sun irradiance. Because of the large number of simulated data points, only some data points are used to represent the results.

### 2.2. Field Experiments

**Figure 2:**
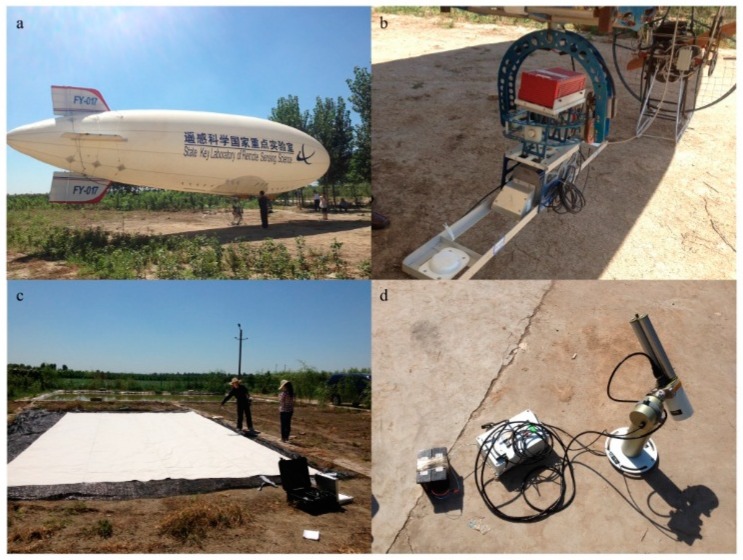
Experimental setup. (**a**) airship; (**b**) AisaEAGLE airborne Hyperspectral Imaging System; (**c**) reference white board; (**d**) CE318.

The airborne experiment was conducted on 1 September 2013 in Baoding, Hebei Province, China. The study site is an agricultural area with maize and trees such as toon and elm, and the mean elevation is 16.8 m. An airborne hyperspectral imaging system designed by Specim Spectral Imaging Oy Ltd. (Oulu, Finland) was carried on the airship (Figure 2a) in this experiment, including an AisaEAGLE sensor, a data acquisition and power unit (DPU), a high-performance GPS/IMU positioning system (POS) and CaliGeoPRO software (Figure 2b). The AisaEAGLE sensor is a high-performance airborne visible and near infrared (VNIR) pushbroom hyperspectral system in the 400–970 nm spectral range with 3.3-nm spectral resolutions. The sensor parameters are listed in Table 3. The POS uses RT3100 produced by Oxford Technical Solutions (city, England), with a positioning accuracy of 0.5 m. The original image was radiometrically and geometrically corrected using CaliGeoPRO software package. In this work, the reference white board (Figure 2c) was set in a blank area in the research area.

The hyperspectral data were acquired from 16:00 to 17:00 UTC + 8 using an AisaEAGLE sensor at a 400-m altitude. At the same time, a high-performance field and laboratory chlorophyll fluorometer (PAM-2500) produced by Heinz Walz GmbH (Effeltrich, Germany) was used to measure the chlorophyll fluorescence parameter on the ground. Several types of leaf samples, which were exposed to direct sunlight, were selected for fluorescence measurements with a measurement time consistent with the flying experiment. The stationary fluorescence from the PAM-2500 measurement results was used to show the vegetation fluorescence in the specific time and light conditions, marked as Fs. The PAM-2500 chlorophyll fluorometer employs a pulse-amplitude-modulated (PAM) measuring light to excite chlorophyll fluorescence. The intensity of PAM excitation light is sufficiently low for monitoring fluorescence yield without affecting the state of photosynthesis. Therefore, PAM-2500 is a popular active technology for measuring chlorophyll fluorescence. Besides, the GPS was used to record the sampling location for localization in the hyperspectral image.

A CE318 photometer (Figure 2d), designed by Cimel Electronique S.A.S (Paris, France) was used to measure the optical properties of the atmosphere. It provided quantification and physical-optical characterization of the aerosols. The SZA, VIS and water content were extracted from the measured data for CE318, and these parameters were then entered into MODTRAN to obtain the atmospheric parameters.

### 2.3. Method of Retrieving SIF

Equation (1) shows that the SIF signal is thought to be an additive term, which is added to the reflect flux in the entire radiative transfer equation at the target level. The at-sensor radiance is the sum of the reflected radiance plus the SIF radiance. Table 4 lists methods used for estimating fluorescence from space-borne data. In this paper, three different methods are used to retrieve SIF: FLD/3FLD [11,12,29,30], DOAS [46] and the method proposed by Braun Raychaudhuri [33], which is referred to as the Braun method. These three methods exploit different strategies to retrieve fluorescence. FLD/3FLD requires accurate atmospheric parameters; DOAS is carried out based on the physical model; and the Braun method uses the no-vegetation region to remove the effects of the atmosphere.

#### 2.3.1. Damm Method

In Equation (1), on the canopy, the effects of atmospheric scattering and absorption are negligible, thus ρ_so_ = 0, Rρ << 1, τ_ss_ + τ_sd_ = 1, and τ_do_ + τ_oo_ = 1, Equation (1) can be simplified as following:

(2) $$L=\frac{E_{g}R}{\pi }+\text{SIF}$$

in which, E_g_ = E_0_cosθ, and the 3FLD principle can be expressed as follows:

(3) $$SIF_{i}=\frac{L_{i}-\frac{E_{g}^{i}\times (w_{left}\times L_{left}+w_{right}\times L_{right})}{w_{left}\times E_{g}^{left}+w_{right}\times E_{g}^{right}}}{1-\frac{E_{g}^{i}}{w_{left}\times E_{g}^{left}+w_{right}\times E_{g}^{right}}}$$

with $w_{left}=\frac{760-753}{771-753},w_{right}=\frac{771-760}{771-753}$. The superscript *i*, which indicates the band inside of the Fraunhofer band, was set to 760 nm, the subscript right was set to 771 nm, and the left band was set to 753 nm.

A more accurate SIF retrieval method should consider atmospheric scattering and absorption processes during data acquisition. Therefore, the 3FLD method and O_2_-A oxygen absorption band are also employed to retrieve SIF [30,48], and this method is marked as Damm method. Two measurements are performed inside of the O_2_-A oxygen-absorption (*i* = 760 nm) band, and the other is performed outside of it (*o* = 753 nm). In this method, the fluorescence and reflectance are assumed to exhibit linear variation inside and outside of the O_2_-A band, not considering the shape of the fluorescence or reflectance spectrum. Based on these two measurements, ${\text{SIF}}_{i}$ can be expressed by the following equation [31,49]:

(4) $$SIF_{i}=B\left[\frac{X_{i}\left(E_{g}^{o}+\Pi \;X_{o}\rho_{o}\right)-AX_{o}\left(E_{g}^{i}+\Pi \;X_{i}\rho_{i}\right)}{B\left(E_{g}^{o}+\Pi \;X_{o}\rho_{o}\right)-A\left(E_{g}^{i}+\Pi \;X_{i}\rho_{i}\right)}\right]$$

in which, $X_{j}=\frac{L_{j}{-L}_{p}^{j}}{\tau_{j}\text{↑}}{,E}_{g}^{j}=\frac{E_{0}^{i}\cos\theta }{\pi }{(\tau }_{\text{ss}}^{j}{+\tau }_{\text{sd}}^{j}),\;j=i,o,$ $A=R_{i}/R_{o}$, $B={\text{SIF}}_{i}/{\text{SIF}}_{o}$. where *i* is inside of the O_2_-A oxygen-absorption band, and o is of outside the O_2_-A oxygen absorption band. A is the factor relating $\rho_{i}$ with $\rho_{o}$, and it is derived from the linear interpolation of ρ of the left and right O_2_-A band shoulders. B is the factor relating ${\text{SIF}}_{i}$ and ${\text{SIF}}_{o}$ (inside and outside of the O_2_-A band), and it is fixed to a value of 0.8 [49,50]. This conclusion about B is also confirmed using the simulated data in this paper by computing the ratio of the internal and external fluorescence:

(5) $$A=\frac{\rho_{758}\omega_{1}+\rho_{771}\omega_{2}}{\rho_{758}},\omega_{1}=\frac{771-760}{771-758},\omega_{2}=\frac{760-758}{771-758}$$

#### 2.3.2. DOAS

Differential optical absorption spectroscopy (DOAS) is a method used to determine concentrations of trace gases by measuring their specific narrow-band absorption structures in the UV and visible spectral regions [51]. DOAS is based on Beer-Lambert’s law of light extinction, and it determines the amount of molecular absorbers along the effective optical light path by fitting and scaling spectra within a given wavelength window [52].

This formula can be expressed as follows:

(6) $$-\ln\frac{L(\lambda ,\theta )}{E_{g}(\lambda ,\theta )}=\sum_{n=1}^{N}\sigma_{n}^{\prime }(\lambda )S_{n}{+\sigma }_{\text{Ray}}{(\lambda )S}_{\text{Ray}}{+\sigma }_{\text{Mie}}{(\lambda )S}_{\text{Mie}}{+\sigma }_{f}{(\lambda )S}_{f}+\sum_{m=1}^{M}a_{m}\lambda_{m}$$

where L(λ) and E_g_(λ) are the measured backscattered radiance and extraterrestrial irradiance, respectively; S_n_ is the number density of either molecules or aerosol particles along the slant optical path; $\sigma_{n}\left(\lambda \right)$ is the absorption cross-section of the n^th^ atmospheric absorber; N is the number of absorbers; $\sigma_{\text{Ray}}\left(\lambda \right)$, $\sigma_{\text{Mie}}\left(\lambda \right)$ and $\sigma_{f}\left(\lambda \right)$ are the reference spectra of Rayleigh scattering, Mie scattering and fluorescence, respectively; and $\sum_{m=1}^{M}a_{m}\lambda_{m}$ is a low-order polynomial, typically of the order *M* < 4. Note that here, $\sigma_{f}\left(\lambda \right)$ is the fluorescence reference spectrum, and it acts as a pseudo-emission across sections in the DOAS method. $S_{f}$ is a DOAS fluorescence fit factor, and it acts as a fluorescence column, representing the pseudo-emission cross-section [46]. Following Khosravi’s research, a 745–758 nm fitting window is selected in this paper. This window seems to be affected only by Fraunhofer lines and not by the deep absorption features of oxygen and water vapor [46], the item $\sum_{n=1}^{N}\sigma_{n}^{\prime }\left(\lambda \right)S_{n}$ can be removed. Rayleigh and Mie scattering are turned to be polynomials with respect to λ. Equation (5) can be simplified as follows:

(7) $$-\ln\frac{I(\lambda ,\theta )}{I_{0}(\lambda ,\theta )}=\sigma_{f}(\lambda )S_{f}+\sum_{m=1}^{M}a_{m}\lambda_{m}$$

M is set to 3. Finally, the coefficients $a_{m}$ and $S_{f}$ are fitted using the least-squares algorithm.

#### 2.3.3. Braun Method

Assume that the effects of the atmospheric parameters are the same in the same district; then, comparing the vegetated region with the non-vegetated region, the effect of atmosphere scattering can be cancelled out, and the fluorescence radiation can be expressed by the following equation [33]:

(8) $$F=(A_{V}-A_{NV})(\omega_{L}L_{L}+\omega_{R}L_{R})$$

in which

(9) $$A_{i}=\frac{\omega_{L}L_{L}^{i}+\omega_{R}L_{R}^{i}-L_{R}^{i}}{\omega_{L}L_{L}^{i}+\omega_{R}L_{R}^{i}},\omega_{L}=\frac{\lambda_{R}-\lambda_{F}}{\lambda_{R}-\lambda_{L}},\omega_{R}=\frac{\lambda_{F}-\lambda_{L}}{\lambda_{R}-\lambda_{L}},i=V,NV$$

where A_V_ is the percentage of reflected radiance inside of the absorption band for vegetation, including the reflected radiance, path radiance and fluorescence radiation; A_NV_ is the contribution of surface reflectance and path radiance due to atmospheric scattering in the absorption band; $L_{L}$ and $L_{R}$ are the radiance on the left and right sides of the oxygen-A band, respectively; $L_{F}$ is the radiance in the oxygen-A band; and $\lambda_{L}$, $\lambda_{F}$ and $\lambda_{R}$ are 758.03 nm, 760.46 nm and 771.01 nm, respectively.

## 3. Results

### 3.1. Sensitivity Analysis

**Figure 3:**
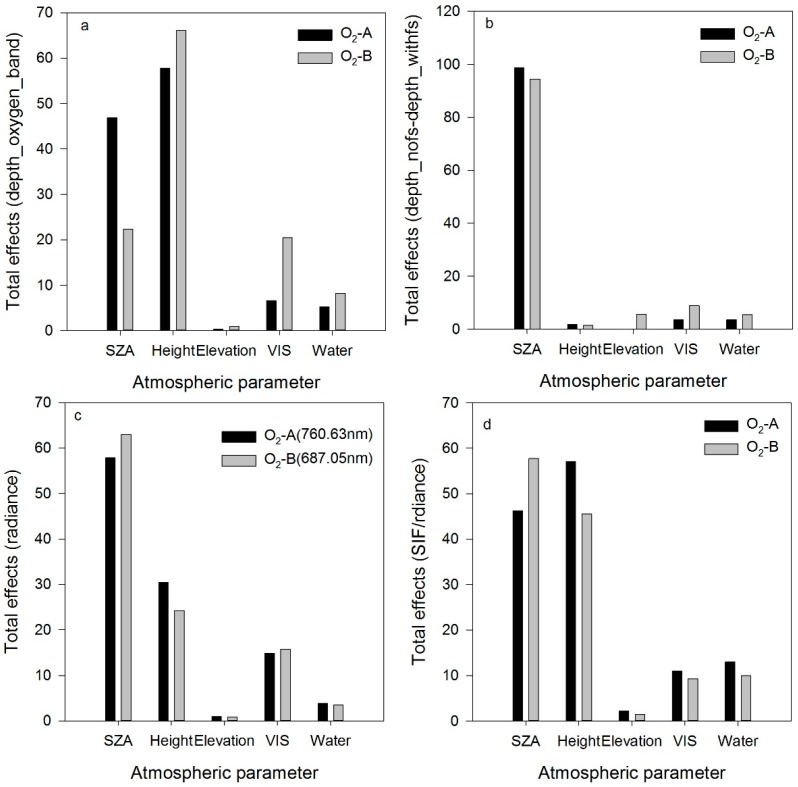
Results of sensitivity analysis. Four indicators are selected: (**a**) depth_oxygen_band; (**b**) depth_nofs-depth_withfs; (**c**) radiance; and (**d**) SIF/radiance.

To illustrate the effects of the atmosphere on TOA radiance, sensitivity analysis was conducted using simulated data to illustrate which atmospheric parameters strongly affected the indicators of fluorescence (Figure 3). Gaussian Emulation Machine for Sensitivity Analysis (GEM-SA) software package was used for sensitivity analysis [53,54]. In sensitivity analysis, the indicator “total effect” was applied to evaluate the effect of each input parameter on the output. In this study, the following four indicators were selected in sensitivity analysis: (1) the depth of the oxygen absorption band, marked as depth_oxygen_band. The absorption band depth was computed by dividing the value for one band outside of the absorption band by that for one band inside of the oxygen absorption band. It is expressed as the ratio a/b, in which a is the radiance outside of the absorption band and b is the radiance inside of it [2,43,55]. In the O_2_-A band, a is determined at 758 nm, and b is measured at 760 nm. In the O_2_-B band, a corresponds to 685 nm and b is measured at 687 nm; (2) the absorption band depth difference. This quantity is computed as the oxygen absorption depth derived from the radiance without the fluorescence minus that including the contribution of fluorescence, marked as depth_nofs-depth_withfs; (3) the radiance including the fluorescence radiance in the O_2_-A band; (4) SIF/radiance (SIF radiance divided by the total radiance). This quantity is used to illustrate the contribution of fluorescence to the total radiance at-sensor in the oxygen absorption band.

Figure 3a shows that the five atmospheric parameters had different effects on the depth of the oxygen absorption band. For the O_2_-A band, the absorption band depth is sensitive to variations in the SZA and sensor height; the effect of elevation can be ignored. The VIS and water content have minor effects on the absorption band depth. In the O_2_-B band, the absorption band depth is the most sensitive to variations in sensor height, and the second most sensitive parameter is the SZA. The VIS and water content follow. The effect of elevation is even weaker and can be ignored.

In the Figure 3b, the absorption band depth difference is used to show the fluorescence. The SZA has a significant effect on the absorption band depth difference in the oxygen absorption bands. The other parameters have a mirror effect and are negligible. Figure 3c shows the effects of variation of the atmospheric parameters on the radiance in the oxygen-absorption band. For the O_2_-A and O_2_-B bands, each parameter has a similar effect on the radiance, and the SZA, sensor height and VIS are much more sensitive than the other parameters. In Figure 3d, the indicator SIF/radiance is selected for analysis. The SZA and sensor height still have strong influences on SIF/radiance. The sensitivity analysis results reveal that the SZA and sensor height are two important atmospheric parameters that affect fluorescence retrieval from space-borne data.

### 3.2. Effects of Atmospheric Parameters on the Oxygen-Absorption Depth in the O_2_-A and O_2_-B Bands

In the oxygen absorption bands or Fraunhofer lines, SIF partially fills the absorption bands by the sun-excited emission of the luminescent target [26]. Because the oxygen absorption bands overlap with the peak of the fluorescence spectrum, they are thought to be the best bands for retrieving fluorescence. By determining the depth of the oxygen absorption band, one can estimate fluorescence qualitatively. The depth and shape of the oxygen-absorption bands are determined by the path length of solar irradiation, and they are affected by, e.g., the solar zenith angle, absorption and scattering of the atmosphere, the surface pressure, the aerosol content, and the water content [2]. Based on the simulated data generated by the MODTRAN and SCOPE models, the effects of atmospheric parameters on the oxygen-absorption depth in the O_2_-A and O_2_-B bands are analyzed in this section.

#### 3.2.1. Solar Zenith Angle

**Figure 4:**
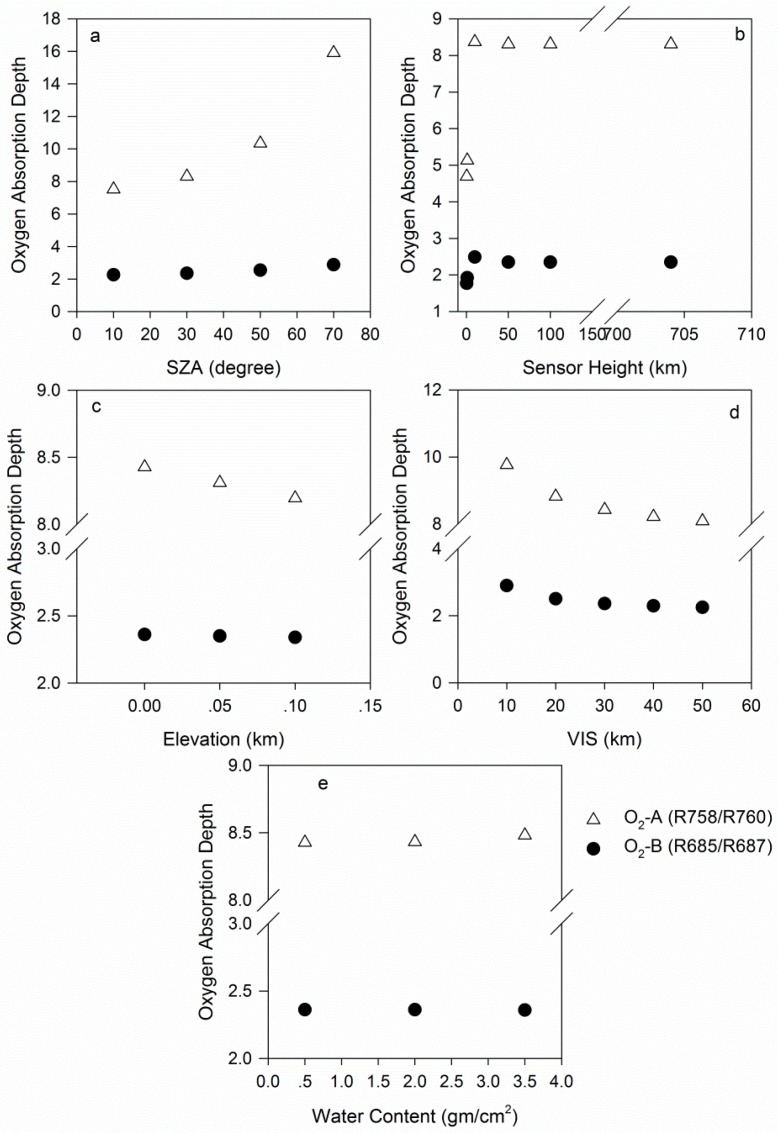
Atmospheric effects on the oxygen absorption band depth.

The SZA causes a strong variation in the path length of solar irradiance. In MODTRAN, the SZA varies from 10° to 70°, with an increment of 20°. The absorption depth in the O_2_-A band is much larger than that in the O_2_-B band. With an increase in the SZA, the oxygen absorption depth increases gradually, but it does so more quickly in the O_2_-A band than in the O_2_-B band (Figure 4a). Because of the variation in the SZA, the observed maximum depth variations are 111.4% in the O_2_-A band and 27.5% in the O_2_-B band (Table 5). The SZA is an important parameter that affects the O_2_-A band absorption depth.

#### 3.2.2. Sensor Height

As shown in Figure 4b, with an increase in sensor height, the oxygen-absorption depth first rapidly increases and then slowly changes. The sensor height is set to vary from 0.5 km to 704 km, including 0.5, 1.0, 10, 50, 100 and 704 km. From 0.5 km to 10 km, the oxygen absorption depth changes quickly, and from 10 km to 704 km, the depths tend to be similar (Figure 4b). These findings are consistent with Damm’s conclusions [30]. The observed maximum depth variations are 77.1% in the O_2_-A band and 32.6% in the O_2_-B band (Table 5).

#### 3.2.3. Elevation

Sensor height and elevation are two factors that determine the geometric air mass and O_2_ absorption rates. Notably, the depth decreases as the elevation increases, particularly in the O_2_-A band (Figure 4c). The variation in elevation from 0.0 to 0.1 km causes depth variations of 2.8% in the O_2_-A band and 0.9% in the O_2_-B band (Table 5).

#### 3.2.4. VIS

VIS is the vertical distribution of the aerosol concentration in MODTRAN, and it characterizes the surface meteorological range. VIS can be expressed as a function of AOD at 550 nm. It varies from a minimum value of 10 km to a maximum value of 50 km, and the depth decreases as VIS increases (Figure 4d). The variation in VIS causes depth variations of 17.2% in the O_2_-A band and 22.4% in the O_2_-B band (Table 5).

#### 3.2.5. Water Content

Figure 4e shows that variation in the water content results in a little change in the oxygen absorption depth (Figure 4e), and that the maximum depth variations are only 0.63% in the O_2_-A band and 0.01% in the O_2_-B band (Table 5). Therefore, the water content is not a sensitive parameter for the depth of either oxygen absorption band. Damm *et al.* [30] reached similar conclusions.

## 4. Discussion

### 4.1. Comparison of the Three Methods of Retrieving Fluorescence

In Section 2.3, three methods of estimating fluorescence are introduced. Based on the simulated data generated in Section 2.1, these three methods are employed to compute fluorescence. The results of these methods are compared with the fluorescence at 761 nm derived from SCOPE (SCOPE SIF), and two indicators are used to assess the performance of these two methods: the correlation parameter (R^2^), and root mean square error (RMSE).

Table 6 shows that the fluorescence values computed using the Damm method and DOAS method in the O_2_-A and O_2_-B bands are strongly correlated with the SCOPE SIF values, whereas the values determined using the Braun method are negatively correlated with the SCOPE SIF values in the oxygen-absorption bands. These findings indicate that marked differences exists between the Braun method and SCOPE SIF. From the Table 6, it can be found that the RMSEs of the three methods are relatively small in the O_2_-A band and are larger for the Damm method and DOAS method in the O_2_-B band. The findings for these three indicators suggest that the Damm method has the best performance, particularly in the oxygen-A band. DOAS is also acceptable for determination of fluorescence, despite the large standard deviation in the oxygen-B band. In contrast, the Braun method cannot be used to estimate fluorescence, as the accuracy and error of the estimated fluorescence may not satisfy our requirements.

The comparisons among the fluorescence values derived from the three methods and SCOPE Fs indicate the Damm method and DOAS method have good retrieval accuracy. The effects of the atmospheric parameters on these two methods are analyzed in Table 7. In this study, the maximum variation is used in analysis and is calculated as the variation value divided by the base value. In the O_2_-A band, the variation of SZA and sensor height cause −9.80% and 2.3% of the fluorescence estimated by the Damm method. The equivalent fluorescence variation values are −0.13 and 0.03 W/m^2^/µm/sr, and the effects of the elevation and water content can be neglected. In the O_2_-B band, the results of sensitivity analysis of the atmospheric parameters are similar for the Damm method. For the DOAS method, VIS is the only sensitive parameter in the O_2_-A band, and the variation in VIS causes a 61.8% fluorescence variation. The effects of SZA, sensor height and elevation are negligible. In the O_2_-B band, elevation is the sensitive parameter, and it causes a variation of 113%. Notably, because of the characteristics of DOAS, in the process of data fitting, the initial value of the model must be given, and the selection of the initial value can result in the abnormal results using this method.

### 4.2. Using the Damm Method to Retrieve Fluorescence from Airborne Imagery

**Figure 5:**
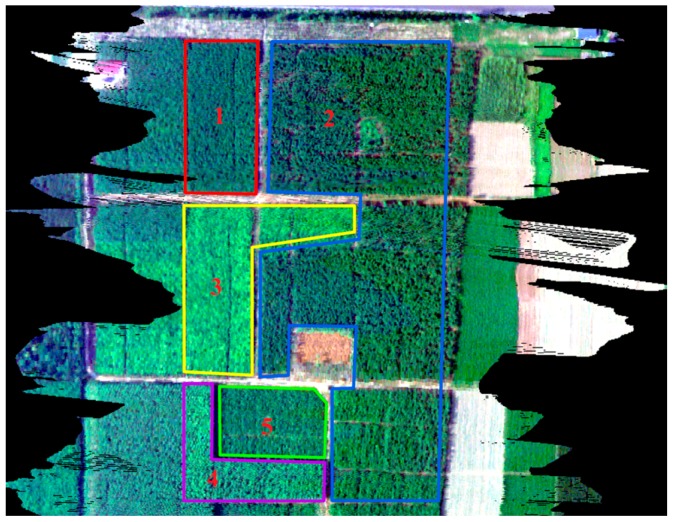
Airborne hyperspectral imagery acquired with an AISA sensor, shown as a true-color composite. Different plants are marked in the figure: 1: ailanthus; 2: elm; 3: mountain peach; 4: willow; and 5: Chinese ash.

The above analysis highlights the importance of atmospheric effects, and it also demonstrates that these effects can be compensated for to obtain accurate SIF measurements using atmospheric absorption features. In this experiment, CE318 is used to measure the atmospheric parameters (see Table 8), and through MODTRAN, the required atmospheric parameters are computed. The Damm method is used to retrieve fluorescence from AISA imagery. Figure 5 depicts the AISA imagery (true-color composite), and five types of vegetation are selected for analysis. The distribution of the vegetation is shown in Figure 5.

**Figure 6:**
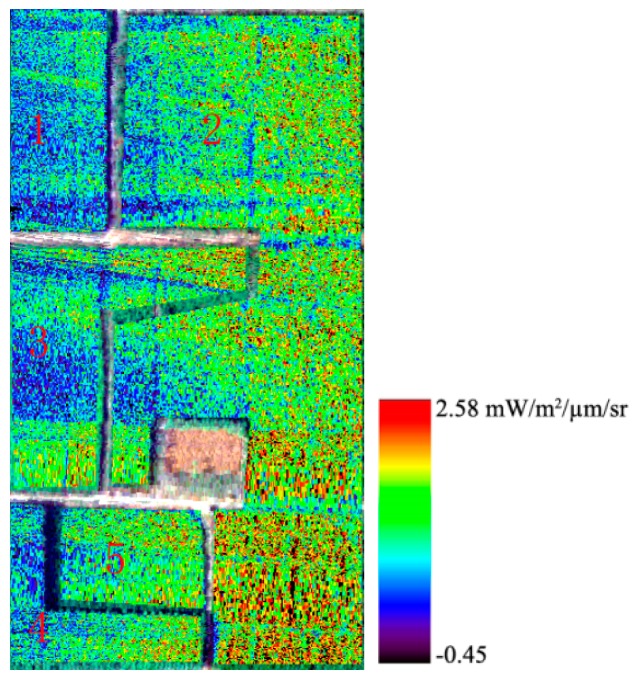
Retrieved fluorescence from AISA imagery using the Damm method with atmospheric information; 1: ailanthus; 2: elm; 3: mountain peach; 4: willow; and 5: Chinese ash.

**Figure 7:**
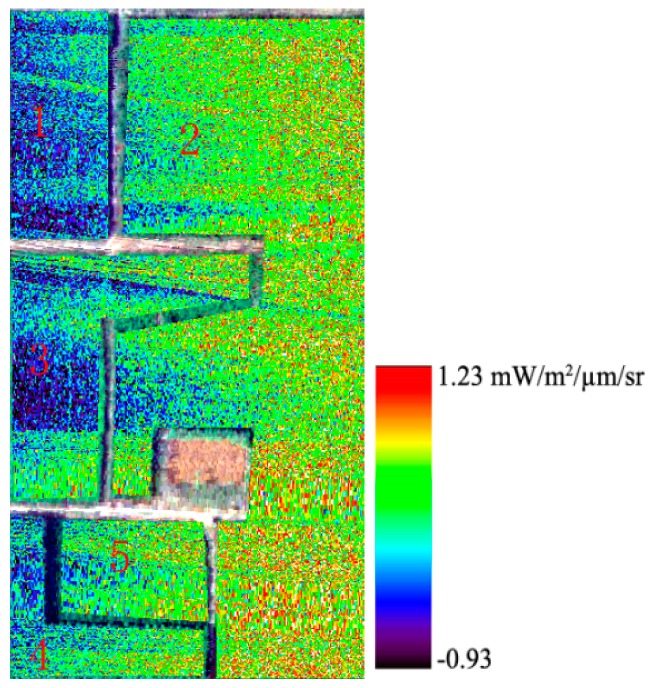
Retrieved fluorescence from AISA imagery using the 3FLD method; 1: ailanthus; 2: elm; 3: mountain peach; 4: willow; and 5: Chinese ash.

The 3FLD and Damm methods are introduced in Section 2.3. In the formulas, L and Eg are the surface-reflected radiance and solar irradiance, respectively. When retrieving fluorescence, L is obtained directly from the hyperspectral image, and Eg is obtained from the reference white board. Figure 6 shows the results of fluorescence retrieval using the Damm method, and Figure 7 presents the fluorescence retrieved using the 3FLD method without considering the atmospheric information. Figure 6 shows that the fluorescence values of elm and Chinese ash are higher than those of the other three types of vegetation and that there are differences among these five types of vegetation. However, it is difficult to draw a clear dividing line between the adjacent vegetation. It is clear that the fluorescence retrieval results are affected by noise. The same conclusions can be made from Figure 7.

**Figure 8:**
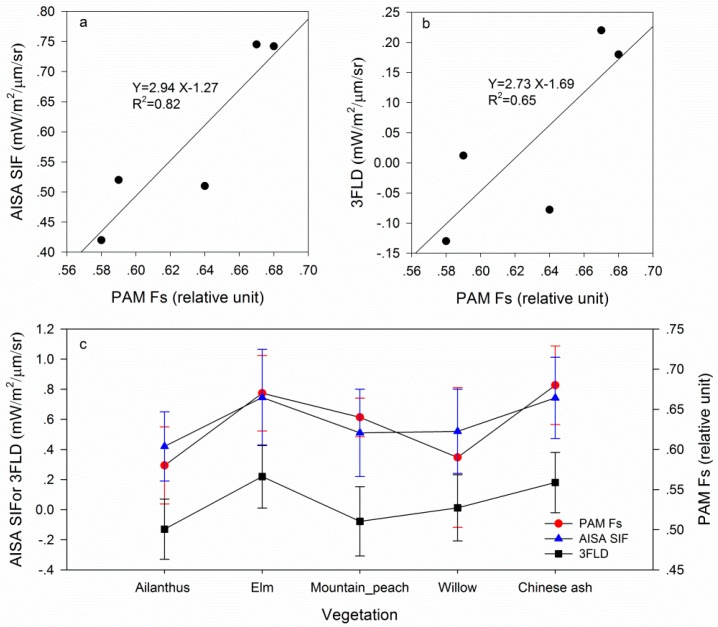
Comparison between AISA SIF and PAM Fs: a is the relationship between AISA SIF and PAM Fs; b is the relationship between 3FLD and PAM Fs; and c is the comparison of fluorescence among the five types of vegetation.

A comparison between Figure 6 and Figure 7 reveals that accurate atmospheric information can improve retrieval accuracy. In the experiment, the PAM is used to measure the steady-state fluorescence of vegetation, whereas the sensor collects data. A comparison between the fluorescence derived from the airborne data and that from PAM-2500 is conducted. R^2^ between the retrieved fluorescence using the Damm method and PAM-2500 is 0.91 (Figure 8a), whereas that between the retrieved fluorescence using the 3FLD method and PAM-2500 is 0.65 (Figure 8b). These results indicate that fluorescence derived from the airborne data is reliable and that results obtained using the Damm method are much closer to the PAM measurements than those obtained using the 3FLD method. Figure 8c shows that there are differences in fluorescence among the different types of vegetation. The mean fluorescence value for each type of vegetation is used herein to illustrate the trend in the variation of vegetation. The results of the PAM measurements demonstrate that the fluorescence of Chinese ash is the highest among the five types of vegetation, followed by elm, mountain peach, willow and ailanthus. The results of the airborne measurement indicate that the fluorescence values of Chinese ash and elm are greater than those of the other vegetation. The trend of variation of the vegetation is consistent between the two measurement scales. These findings also indicate that it is possible to detect different types of vegetation using fluorescence.

## 5. Conclusions

Remote sensing of fluorescence from space is currently an important research subject, and it is the core mission objective of ESA FLEX. Preliminary research regarding space-based retrieval of fluorescence has been reported in the literature. Several methods for estimating fluorescence have been developed and applied to GOSAT (TANSO-FTS sensor), MetOp (GOME-2), Envisat (SCIMACHY) and OCO-2 images. Fluorescence retrieval can be performed in the oxygen-absorption bands overlapping the peaks of fluorescence emission using high-spectral resolution data. Combined with atmospheric information, it is feasible to decouple the fluorescence flux from the reflected solar radiation accurately.

The effects of the atmosphere are no negligible in the method of retrieving fluorescence. Therefore, in this paper, we first study the effects of the atmospheric parameters on fluorescence in oxygen-absorption bands using simulated data generated by MODTRAN4 and SCOPE. Under the different combinations of canopy (Cab, Fqe and LAI), atmospheric (VIS and water content) and imaging geometric parameters (SZA, sensor height and elevation), the TOA radiance, including the contribution of fluorescence, is simulated by the radiation transfer equation. We test the sensitivities of five parameters (SZA, sensor height, elevation, VIS and water content) to the oxygen absorption depth and other indicators relative to fluorescence. Then, we examine the accuracies of the Damm, Braun and DOAS methods for fluorescence retrieval and the performances of these three methods under variation of the atmospheric parameters. Finally, we apply the Damm method to the AISA airborne image and compare airborne fluorescence with ground measurements.

In the process of sensitivity analysis, we select four indicators to test the sensitivity to the atmosphere in the O_2_-A and O_2_-B bands: depth_oxygen_band, depth_nofs-depth_withfs, radiance and SIF/radiance. The results show that SZA, sensor height and VIS are the significant and sensitive parameters of these four indicators. In the following analysis, we focus on the sensitivity of the atmospheric parameters on the oxygen absorption depth. The depth increases gradually with increases in SZA and sensor height. In contrast, it decreases with increases in VIS and elevation, and it changes little with variation in water content.

Analysis of Damm method, Braun and DOAS shows that the Damm and DOAS methods can be used to retrieve SIF; in contrast, the Braun method has a large error. The Damm and DOAS methods yield good results with SCOPE SIF. The Damm method is sensitive to variations in SZA and sensor height in the O_2_-A and O_2_-B bands. For the DOAS method, elevation and VIS are sensitive parameters in the O_2_-A and O_2_-B bands, respectively.

A comparison of these three methods shows that accurate atmospheric parameters can improve the fluorescence retrieval accuracy. We use the Damm and 3FLD methods to retrieve fluorescence from an AISA airborne image, and we analyze the results of these two methods. Furthermore, we compare the results of these two methods with the PAM-2500 measurements. The results reveal that the retrieval fluorescence has a strong relationship with the PAM-2500 measurements and that the performance of the Damm method is better than that of the 3FLD method. Analysis of five plants, *i.e.*, ailanthus, elm, mountain peach, willow and Chinese ash, revealed the presence of differences in fluorescence among the different types of vegetation. Among these five types of vegetation, the fluorescence values of Chinese ash and elm are greater than those of the other vegetation.

In this paper, the simulated data are noise-free, and the influence of the Ring effect, which can result in uncertainty in fluorescence retrieval, is neglected. Future research regarding fluorescence retrieval will delve deeper and consider more complex conditions.

## Figures and Tables

**Table 1:** Input parameters of MODTRAN used in the generation of simulated data.

Parameter	Value	Unit	Description
SZA	10, 30, 50,70	Degree	Sun zenith angle
Sensor height	0.5, 1.0, 10, 50, 100, 704	km	Position of sensor
Elevation	0.0, 0.05, 0.1	km	Altitude of surface relative to sea level
VIS	10, 20, 30, 40, 50	km	Surface meteorological range
Water content	0.5, 2.0, 3.5	gm/cm^2^	Vertical water vapor column

**Table 2:** Input parameters of SCOPE in this paper.

Parameter	Value	Unit	Description
Cab	20, 40, 60, 80	µg/cm^2^	Chlorophyll *α* + *b* content
Fqe	0.02, 0.04, 0.06	--	fluorescence quantum yield efficiency
LAI	1, 2, 4, 6	m^2^/m^2^	leaf area index

**Table 3:** The AisaEAGLE sensor parameters.

Parameter	Value
Spectral range	400–970 nm
Spectral resolution	3.3 nm
Spectral sampling interval	0.67 nm
Focal length	18.5 mm
FOV	36.7 degrees
IFOV	0.036 degrees
Swath width	0.66 × altitude
Ground resolution @ 400-m altitude	0.32 m
SNR	1250:1 (maximum theoretical)

**Table 4:** Review of the methods for retrieving fluorescence from space-borne data.

Reference Paper	Band	Method	Application
**Fraunhofer lines**			
Joiner *et al.* (2011) [24]	769.9–770.25 nm (K I)	${\left(L_{\text{TOA}}\right)}^{*}={\left(\frac{RE_{0}\cos\theta }{\pi }+F_{s}\right)}^{*}=KE^{*}+F$	GOSAT TANSO-FTS
Joiner *et al.* (2012) [35]	769.9–770.25 nm 758.45–758.85 nm 863.5–868.5 nm (Ca II)	${\left(L_{\text{TOA}}\right)}^{*}={\left(\frac{RE_{0}\cos\theta }{\pi }+F_{s}\right)}^{*}=KE^{*}+F$	GOSAT SCIAMACHY
Guanter *et al.* (2012) [36]	755–775 nm (K I)	$F\left(\omega ,F_{S}\right)=\sum_{i=1}^{n_{v}}\omega_{i}v_{i}+F_{S}^{TOA}I$	GOSAT-FTS
N. Khosravi (2012) [46]	660–683 nm 745–758 nm	DOAS	
Guanter *et al.* (2013) [37]	745–759 nm (Fraunhofer line) 717–759 nm (red edge) 745–780 nm (O_2_-A band) 717–780 nm (full-range)	$F\left(a,b,c,\;F_{s}{}^{760}\right)=v_{1}\sum_{i=1}^{n_{p}}a_{i}\lambda^{i}+v_{2}\sum_{i=1}^{n_{p}}b_{i}\lambda^{i}+\sum_{i=1}^{n_{p}}c_{i}v_{i}+F_{s}{}^{760}h_{F}$	GOSAT-FTS HR4000
P. Köhler *et al.* (2014) [38]	590–790 nm (GOME-2) 604–805 nm( SCIAMACHY)	$L_{\text{TOA}}=I_{sc}\cdot \frac{\mu_{0}}{\pi }\cdot \sum_{i}\left(a_{i}\cdot \lambda^{i}\right)\cdot \sum_{j}\left(\beta_{j}\cdot PC_{j}\right)+F_{s}\cdot h_{f}\cdot T_{↑}$	GOME-2 SCIAMACHY
P. Köhler *et al.* (2015) [39]	755–759 nm	GARLiC	GOSAT
**Oxygen-absorption band**			
Guanter *et al.* (2007) [12]	760.6 nm 753.8 nm	FLD MODTRAN-4	MERIS CASI-1500
Damm *et al.* (2010) [11]	760.6 nm 755 nm	FLD MODTRAN-4	ASD
Guanter *et al.* (2010) [29]	745–775 nm 672–702 nm	SFM FLD-S	FIMAS-like TOA radiance
Mazzoni *et al.* (2010) [47]	677–697 nm 750–770 nm	DS = NSENSOR_RADn-NSENSOR_RADm	OCO TANSO-FTS
Frankenberg *et al.* (2011) [31]	O_2_-A	$\vec{f}\left(F_{s}{}^{rel},\;a\right)=\log\left(\langle \vec{I_{0}}+F_{s}{}^{rel}\rangle \right)+\sum_{i=0}^{n}a_{i}\cdot \lambda^{i}$	GOSAT OCO-2
Joiner *et al.* (2013) [32]	715–745 nm 750–780 nm	$\rho_{tot}\left(\lambda \right)=\rho_{s}\left(\lambda \right)Τ\left(\lambda \right)\bar{Τ}\left(\lambda \right)+\frac{\pi F_{s}\left(\lambda \right)\bar{\bar{Τ}}\left(\lambda \right)}{E\left(\lambda \right)\cos\theta_{0}}$	GOME-2
Damm *et al.* (2014) [30]	O_2_-A	3FLD MODTRAN-5	ASD
Braun (2014) [33]	O_2_-A	F = A_V_ -A_NV_	EO-1
Liu *et al.* (2015) [34]	650–800 nm	F-SFM	simulated data

**Table 5:** Sensitivity analysis. The depth variation is the maximum variation and is calculated as the variation value divided by the base value.

Parameter	Variation Range	Correlation with Depth	Depth Variation	
			O_2_-A	O_2_-B
SZA	10–70	+	111.4%	27.5%
Sensor height	0.1–704 km	+	77.1%	32.6%
Elevation	0.0–0.1 km	-	2.80%	0.90%
VIS	10–50 km	-	17.2%	22.4%
Water content	0.5–3.5 gm/cm^2^	+	0.63%	0.01%

**Table 6:** Comparison of Damm method, Braun and DOAS methods.

Methos *vs.* SCOPE SIF	Fitting Window			
	O_2_-A Band		O_2_-B Band	
	*R*^2^	RMSE	*R*^2^	RMSE
Damm *vs.* SCOPE SIF	0.99	0.13	0.88	0.84
Braun *vs.* SCOPE SIF	−0.20	1.37	−0.73	5.31
DOAS *vs.* SCOPE SIF	0.78	0.40	0.66	1.58

RMSE unit: W/m^2^/μm/sr.

**Table 7:** Study of the sensitivities of different methods to atmospheric parameters. The variation is the observed maximum fluorescence variation, and it is calculated as the variation value divided by the base value. ΔF is the max value of variation in fluorescence. The reference configuration is for SZA = 30°, sensor_height = 704 km, elevation = 0.0 km, VIS = 30 km and water content = 0.5 gm/cm^2^. To assess the performance of one parameter, the other parameters are set as a reference configuration.

Indicator	Band	Method	SZA	Sensor Height	Elevation	VIS	Water Content
			10°–70°	0.5–704 km	0.0–0.1 km	10–50 km	0.5–3.5 gm/cm^2^
Variation	O_2_-A	Damm	−9.80%	2.30%	0.00%	−0.06%	0
		DOAS	0.00%	0.00%	0.00%	61.80%	0.44%
	O_2_-B	Damm	19.40%	13.50%	0.12%	0.62%	0.41%
		DOAS	0.03%	0.12%	113%	0.66%	0.01%
ΔFW/m^2^/µm/sr	O_2_-A	Damm	−0.13	0.03	0.0004	−0.0008	0
		DOAS	−0.0003	−0.00018	0	−0.74	0
	O_2_-B	Damm	−0.88	0.49	0.005	0.03	−0.02
		DOAS	0	0.004	−3.9	−0.002	0

**Table 8:** The retrieved parameters by CE318.

Parameter	Value	Unit
SZA	30.10	degree
Water content	3.97	gm/cm^2^
VIS	44.48	km

## Abbreviations:

SIF	solar-induced chlorophyll fluorescence
Fs	fluorescence measured by PAM-2500
FLEX	Fluorescence Explorer
TOA	top of atmosphere
VNIR	visible and near-infrared
SZA	sun zenith angle
VIS	visibility
FLD	Fraunhofer line depth
DOAS	differential optical absorption spectroscopy
Cab	chlorophyll a+b content
Fqe	fluorescence quantum yield efficiency
LAI	leaf area index
SCOPE Fs	fluorescence radiance at 761 nm
